# The effects of very high fat, very low carbohydrate diets on safety, blood lipid profile, and anabolic hormone status

**DOI:** 10.1186/1550-2783-11-S1-P39

**Published:** 2014-12-01

**Authors:** Jeremy Silva

**Affiliations:** 1 The University of Tampa, Tampa, Florida, USA

## Background

Very low carbohydrate (<5 %), high fat (>70 %) (VLCKD) diets have previously been shown to decrease fat mass in obese or overweight individuals. The very high fat, high cholesterol diet has caused alarm and researchers question its safety. However, because lipids provide the raw substrate necessary for the biosynthesis of anabolic hormones such as testosterone the diet may have implications for athletes. PURPOSE: Therefore the purpose of this study was to investigate the effects of 11 weeks of VLCKD dieting on safety, blood lipid profile, and anabolic hormone status.

## Methods

Twenty-six college aged resistance trained men volunteered to participate in this study and were divided into VLCKD (5 % CHO, 75 % Fat, 20 % Pro) or a traditional western diet (55 % CHO, 25 % fat, 20 % pro). All subjects participated in a periodized resistance-training program three times per week. Blood samples were taken at week 0 and 11 of the study and analyzed for safety (comprehensive metabolic panel, and comprehensive blood panel), blood lipid profile (triglycerides, HDL, LDL and total), and insulin and testosterone. Consent to publish the results was obtained from all participants.

## Results

There were no differences in any of the safety parameters measured (CBC / CMP) in either the VLCKD or traditional group. Total cholesterol increased slightly in the VLCKD group while it decreased in the traditional western group. However this rise was driven by an increase in HDL in the VLCKD group (6.69 mg/dl) compared to the western (-1.6 mg/dl) with no changes in LDL. Triglycerides were significantly higher in the VLCKD group (29.3 mg/dl) than the western (-8.4 mg/dl). Total testosterone increased significantly in the VLCKD diet (118 ng/dl) as compared to the western (-36 ng/dl) while insulin increased significantly in the western group (3.7 uIu/ml) compared to the VLCKD (.1 uIu/ml).

## Conclusion

This study data suggests that a VLCKD is safe. In addition this diet strategy improves testosterone values while simultaneously increasing insulin sensitivity. Lastly, even though the total cholesterol increased in the VLCKD group, their HDL drove this response. Athletes looking to optimize their hormone levels while employing a safe dieting strategy can use a VLCKD.

